# Effects of romosozumab combined with routine therapy on pain relief, disease progression and adverse reactions in patients with postmenopausal osteoporosis: a systematic review and meta-analysis

**DOI:** 10.3389/fmed.2024.1440948

**Published:** 2024-08-14

**Authors:** Ge Gao, Jian Cui, Yuanyuan Xie, Jing Dong

**Affiliations:** ^1^Central Hospital Affiliated to Shandong First Medical University, Jinan, China; ^2^The First Affiliated Hospital of Shandong First Medical University (Shandong Qianfo Mountain Hospital), Jinan, China

**Keywords:** romosozumab, menopause, osteoporosis, fracture, adverse reactions

## Abstract

**Background:**

Postmenopausal osteoporosis (PMOP) increases fracture risk in women. Though traditional treatments are slow to act, combining romosozumab with conventional therapy shows promise. Despite its growing use, studies on effectiveness are limited. This study aims to systematically evaluate the combined therapy’s impact on pain relief, disease progression, and adverse reactions in PMOP patients.

**Methods:**

Databases including PubMed, EMBASE, ScienceDirect, and the Cochrane Library were searched from their inception to September 2023 to identify randomized controlled trials (RCTs) evaluating the role of romosozumab in PMOP. Random or fixed effect models were employed for statistical analysis. Two reviewers independently assessed the quality of the included studies and extracted the data. The meta-analysis was conducted using RevMan 5.4 software.

**Results:**

Six RCTs with a total sample size of 17,985 cases were included. The incidence of vertebral fractures was compared and analyzed after 12 and 24 months of treatment. Romosozumab significantly reduced the incidence of vertebral fractures at 24 months (OR = 0.36; 95% CI: 0.35–0.52) but not at 12 months (OR = 0.39; 95% CI: 0.14–1.05). It was also associated with a decreased incidence of nonvertebral fractures (OR = 0.79; 95% CI: 0.66–0.94) and clinical fractures at 24 months (OR = 0.70; 95% CI: 0.59–0.82) compared to standard therapy. Romosozumab demonstrated a significant improvement in percentage change in bone mineral density (BMD) [mean difference (MD) = 10.38; 95% CI: 4.62–16.14] and in hip joint BMD (MD = 4.24; 95% CI: 2.92–5.56). There was no notable difference in adverse reactions compared to standard care (*p* > 0.05). Funnel plots displayed a predominantly symmetrical pattern, suggesting no evidence of publication bias in the selected literature.

**Conclusion:**

Combining romosozumab with conventional therapy effectively treats PMOP, significantly reducing vertebral, non-vertebral, and clinical fractures while increasing BMD in the hip, femoral neck, and lumbar spine. However, further high-quality studies are needed for validation.

## 1 Introduction

Postmenopausal osteoporosis (PMOP) is the most common form of primary osteoporosis, typically characterized by high bone turnover. PMOP is age-related and generally occurs within 5–10 years after menopause. The cumulative bone loss rate during early menopause is higher than that during late menopause. The occurrence of PMOP is mainly affected by the sharp decrease in estrogen levels in the postmenopausal body, which results in greater bone resorption than bone formation. This leads to reduced bone mass, changes in bone structure, bone pain, decreased height, brittle fractures, and other symptoms (1, 2). Osteoporosis (OP) is an asymptomatic or “silent” disease that represents a significant and growing economic burden on healthcare systems and societies worldwide. PMOM greatly increases the likelihood of fragility fractures in about 50% of women. These fractures can have severe and disabling effects, resulting in long-lasting discomfort and restrictions in physical abilities, ultimately leading to a reduced quality of life (3).

The China OP prevalence study revealed that the prevalence of OP among postmenopausal women was 32.1%, significantly higher than the prevalence among men (4). It is found that the prevalence level of OP in women over 50 years old in China is remarkably higher than that in the United States, Canada, and other countries (5, 6). OP patients experience varying degrees of decline in BMD and bone mass, decreased bone strength, and an increased risk of brittle fractures. According to relevant epidemiological data from the United States, the risk of fractures in female OP patients is even higher than the combined risk of breast, ovarian, and uterine cancers (7). Furthermore, osteoporotic fracture patients are more likely to experience recurrent fractures. Studies have shown that patients who have suffered hip fractures are at 2.5 times higher risk of recurrent fractures, while patients with vertebral fractures have a 4 times higher risk. Additionally, patients who have suffered other types of fractures also face a higher risk of recurrent fractures, estimated to be approximately 2–3 times higher (8, 9). Preventing, diagnosing, and treating OP is crucial as it has a remarkable impact on the life quality of patients and leads to a high incidence of osteoporotic fractures.

In addition to vitamin D and calcium as basic treatments, drugs to treat OP are classified into two main categories based on their mechanism of action. The first category includes drugs that inhibit bone resorption, such as calcitonin, estrogen receptor modulators, bisphosphonates, and receptor activator of NF-κB (RANKL) inhibitors. The second category includes drugs that promote bone formation, such as parathyroid hormone analogues and parathyroid hormone-related peptide analogues (10). In January 2018, the European Medicines Agency (EMA) approved the listing of romosozumab (EVENITY^™^), which was jointly developed by Amgen and UCB. The US Food and Drug Administration (FDA) approved the product for marketing in April 2019. Romosozumab is a monoclonal antibody that has been humanized, and it functions by inhibiting the activity of sclerostin, a protein that negatively regulates bone metabolism. This inhibition can promote both bone formation and bone resorption. The treatment is accessible to postmenopausal women at high risk of fractures, as well as patients who cannot tolerate other medications. Romosozumab holds the distinction of being the world’s first sclerostin inhibitor approved for marketing, and it is currently the sole OP drug with dual effects (11, 12).

Several clinical controlled studies have been conducted to explore the therapeutic effects of romosozumab on patients with PMOP. However, the conclusions of these studies vary, and significant differences exist in their designs, leading to poor applicability (13). The current literature on the clinical efficacy of romosozumab in treating PMOP provides inconclusive results. Therefore, romosozumab’s efficacy in treating PMOP should be evaluated through high-quality research. Additional research is needed to establish the efficacy of romosozumab in combination with conventional therapies. More high-quality scientific studies are necessary to provide reliable evidence on the feasibility of this approach. Hence, this study aims to conduct a meta-analysis to analyze the effectiveness of romosozumab in combination with conventional therapy for postmenopausal patients with OP. The study seeks to offer new insights for clinical advancements.

## 2 Methods

This systematic review and meta-analysis were performed in compliance with the Preferred Reporting Items for Systematic reviews and Meta-Analyses (PRISMA) guidelines.

### 2.1 Search strategy

We conducted a comprehensive search for relevant studies on PMOP across various databases, including PubMed, EMBASE, ScienceDirect, and the Cochrane Library. Relevant information regarding the treatment of PMOP with romosozumab was collected. A literature search was conducted using the combination of the following medical subject headings with the Boolean operators: romosozumab, menopause, OP, disease progression, and clinical prognosis.

### 2.2 Eligibility criteria

#### 2.2.1 Literature inclusion criteria

Population/participants: Patients diagnosed with OP using dual-energy X-ray absorptiometry (DXA) measurements of bone mineral density (BMD) in one of three ways:

- BMD *T*-score of ≤ −2.5 at the L1-4 lumbar spine, femoral neck, total hip, or distal radius 1/3.
- History of brittle fracture occurring in the vertebral body or hip.
- *T*-score between −2.5 and −1.0 (indicating low bone mass) accompanied by a history of brittle fracture in the proximal humerus, pelvis, or distal forearm.

Intervention: Romosozumab based routine treatment.

Comparison: Placebo.

Outcome: Studies that reported multiple outcomes, including vertebral fracture rate, non-vertebral fracture rate, clinical fracture rate, lumbar BMD, hip joint BMD, femoral neck BMD, and the incidence of adverse reactions.

Study design: Randomized controlled trials (RCT) evaluating the efficacy of romosozumab in combination with standard treatment for PMOP.

#### 2.2.2 Exclusion criteria

Retrospective cohort studies, case-control studies, case series, reviews, case reports, meta-analyses, as well as *in vitro* and animal studies were not considered for inclusion.

### 2.3 Quality assessment and data extraction

The study included an evaluation of bias risk, which was performed using the bias risk assessment tool recommended by the Cochrane Systematic Review Manual 5.3.2. Two researchers independently screened the literature and extracted data, while also assessing the quality of the extracted data and cross-checking it. Any discrepancies were resolved through discussion or by consulting a third researcher. Express document management software and Excel office software were used to manage and extract research data. If the study provided incomplete data, we contacted the authors of articles. The extracted data included author name, publication date, number of cases, study methods, and outcome indicators such as vertebral fracture rate, non-vertebral fracture rate, clinical fracture rate, lumbar BMD, hip BMD, femoral neck BMD, and incidence of adverse events.

### 2.4 Data synthesis and statistical analysis

Meta-analysis was conducted using RevMan 5.4 software from the Cochrane Collaboration Network. A two-tailed *p* < 0.05 was considered statistically significant. Relative risk (RR) was employed as the effect index. The average and standard deviation values of lumbar BMD, hip joint BMD, and femoral neck BMD were entered into RevMan 5.4, with weighted mean difference (WMD) used as the effect index. A 95% confidence interval (CI) was calculated for all analyses. To assess the heterogeneity among studies, the *χ*^2^ test was performed initially. Studies with *p*-values greater than 0.05 and *I*^2^ values less than 50% were deemed homogeneous, prompting the adoption of a fixed-effect model for the meta-analysis. In cases where the *p*-value was less than 0.05 and the *I*^2^ value equaled or exceeded 50%, indicating a significant level of heterogeneity among studies, the random-effects model was utilized to ascertain the combined effect and evaluate the study’s homogeneity. When the *p*-value was less than 0.05 and the source of heterogeneity remained unclear, a descriptive analysis was employed instead of a meta-analysis.

## 3 Results

### 3.1 Literature search

**Figure 1:**
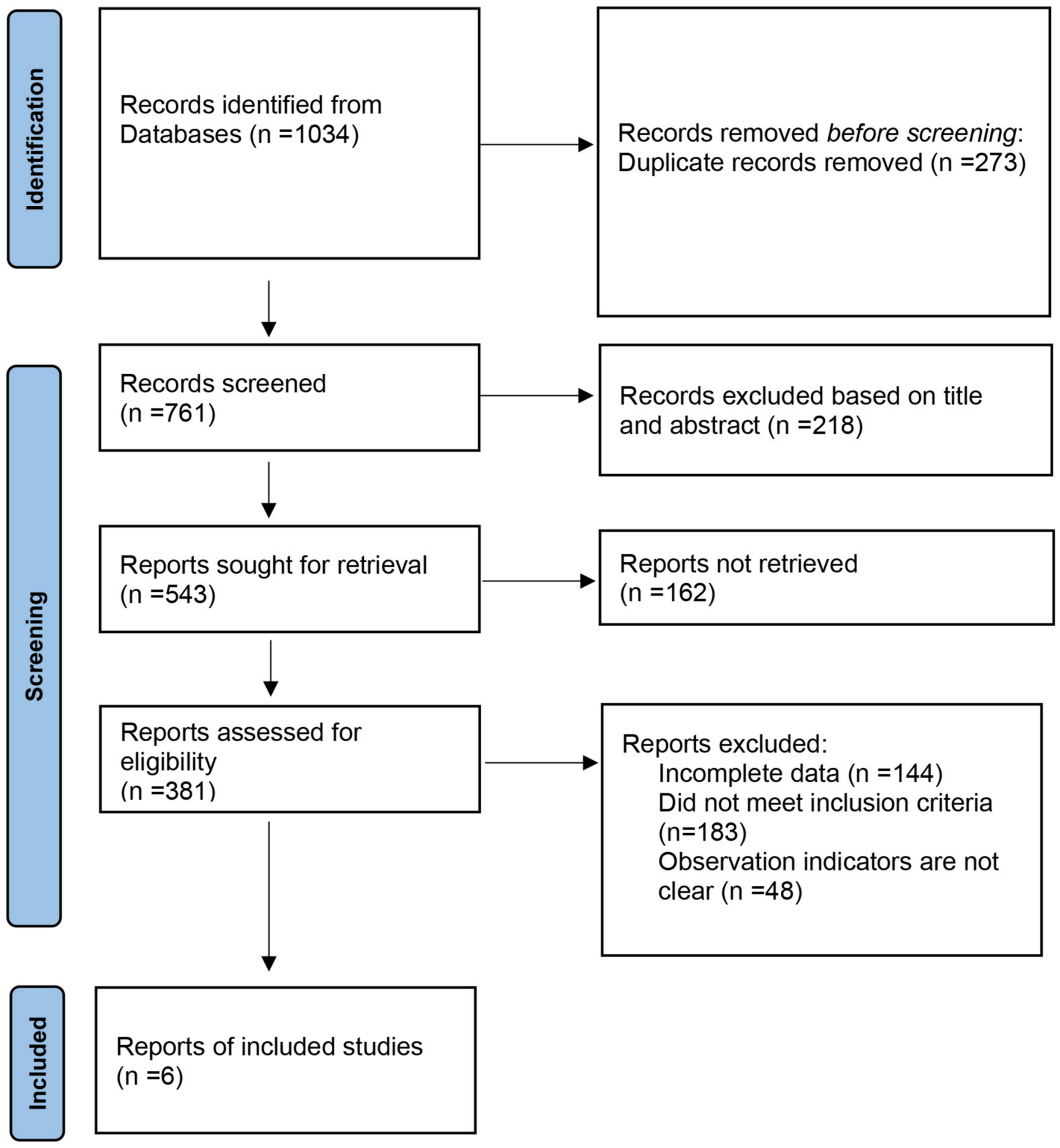
Flowchart of the literature screening process.

Following the Preferred Reporting Items for Systematic Reviews and Meta-Analyses (PRISMA) guidelines, a literature study was conducted. The database search yielded 1,034 articles, and after removing duplicates, 761 articles remained. Upon reviewing titles and abstracts, 543 articles were identified, with 387 articles being excluded due to their irrelevance, being reviews, case reports, or lacking controlled literature. Subsequently, the full texts of the remaining articles were carefully reviewed, and incomplete data were excluded. Six randomized controlled trials (RCTs) were ultimately included, comprising a total of 17,985 participants for meta-analysis (14–19). The flowchart illustrating the literature screening process is displayed in Figure 1. Table 1 presents the characteristics of the included studies.

**Table 1:** Basic characteristics of included studies.

Include the literature	Year of publication	Sample size		Intervention measures		Age (years)	*T*-score of hip joint or femoral neck	Outcome index
		Control group	Research group	Control group	Research group			
Cosman et al. (14)	2016	3,322	3,591	Placebo treatment	Romsozumab plus routine therapy	Unknown	−2.5 to 3.5	① ② ③
Langdahl et al. (15)	2017	209	206	Routine treatment	Romsozumab plus routine therapy	55–90	−2 ± 5	④ ⑤ ⑥
Inshibashi et al. (16)	2017	59	59	Placebo treatment	Romsozumab plus routine therapy	55–85	≤ −2.5	④ ⑤ ⑥
Saaga et al. (17)	2017	2,047	2,046	Routine treatment	Romsozumab plus routine therapy	Unknown	Unknown	① ② ③
McClung et al. (18)	2014	47	100	Routine treatment	Romsozumab plus routine therapy	55–85	≤ −2.0	④ ⑤ ⑥
Cosman et al. (19)	2018	3,148	3,151	Routine treatment	Romsozumab plus routine therapy	Unknown	Unknown	④ ⑤ ⑥

① Bone fracture rate of vertebral body. ② Non-vertebral bone fracture rate. ③ Clinical bone fracture rate. ④ Changes of bone mineral density of lumbar vertebrae. ⑤ Changes of bone mineral density of hip joint. ⑥ Density change of femoral neck.

### 3.2 Quality assessment

In this meta-analysis, three out of the six studies reported detailed patient baseline characteristics. All literature included in this study provided comprehensive descriptions of the observational indicators and research methods employed, along with specific grouping methods. Additionally, they reported on the implementation of blinding, including the number and reasons for its use, as well as any loss of follow-up or withdrawal during the study period. Based on the analysis of the Jadad scale, studies with a score of ≥3 was considered high-quality, while that with a score of ≤2 was considered low-quality. Supplementary Figures S2, S3 display the risk bias analysis.

### 3.3 Meta analysis result

#### 3.3.1 Bone fracture rate of vertebral body

**Figure 2:**
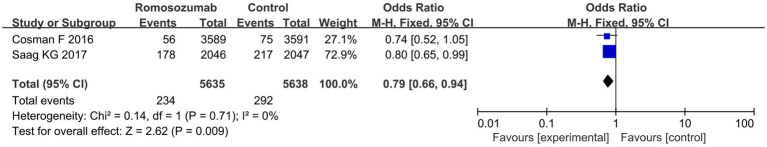
Comparison of vertebral fracture rate between the two groups after treatment.

Two studies reported vertebral fractures after 12 and 24 months of treatment. The pooled results indicated a significant decrease in the incidence of vertebral fractures at 24 months (OR = 0.36; 95% CI = 0.35–0.52, *p* < 0.00001; *I*^2^ = 87%) but not at 12 months (OR = 0.39; 95% CI: 0.14–1.05, *p* = 0.06; *I*^2^ = 90%) (Figure 2). According to the results of heterogeneity test, Chi^2^ = 17.87, df = 3, *p* = 0.0005, *I*^2^ = 83%, indicating that heterogeneity was evident in the research data, which was analyzed by random effect model (Figure 2).

#### 3.3.2 Non-vertebral bone fracture rate

**Figure 3:**
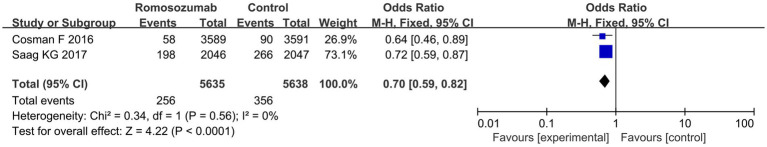
Comparison of non-vertebral bone fracture rate between the two groups after treatment.

Two studies reported the incidence of non-vertebral fractures in patients after treatment. The meta-analysis results showed that romosozumab was associated with a decreased incidence of nonvertebral fractures (OR = 0.79; 95% CI = 0.66–0.94, *p* = 0.009; *I*^2^ = 0%) (Figure 3). According to the results of the heterogeneity test, Chi^2^ = 0.14, df = 1, *p* = 0.71, *I*^2^ = 0%, indicating that the included research data exhibited no significant heterogeneity (Figure 3).

#### 3.3.3 Clinical fracture scale

**Figure 4:**
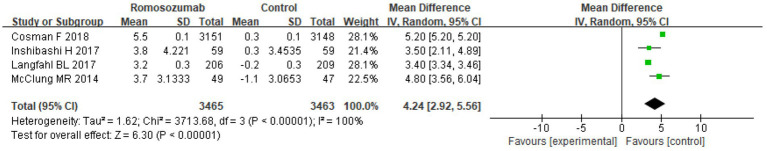
Comparison of clinical bone fracture rate between the two groups after treatment.

Two studies reported the incidence of clinical fractures in patients after treatment. Pooled results revealed that, compared to standard therapy, romosozumab decreased clinical fractures at 24 months (OR = 0.70; 95% CI = 0.59–0.82, *p* < 0.00001; *I*^2^ = 0%) (Figure 4). According to the results of the heterogeneity test, Chi^2^ = 0.34, df = 1, *p* = 0.56, *I*^2^ = 0%, indicating that the included research data showed no significant heterogeneity.

#### 3.3.4 Percentage change in BMD of lumbar vertebra

**Figure 5:**
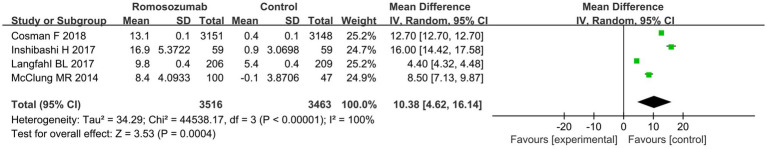
Forest plot comparing lumbar BMD between two groups after 12 months of treatment.

Percentage BMD change at the lumbar spine in the romosozumab versus control group at 12 months was analyzed in four studies. Four RCTs, comprising a total of 3,516 patients in the romosozumab group and 3,463 patients in the standard care group, demonstrated significant improvement in percentage change BMD with romosozumab [mean difference (MD) = 10.38; 95% CI = 4.62–16.14, *p* = 0.0004; *I*^2^ = 100%] (Figure 5). Heterogeneity test results showed significant heterogeneity among the included studies, with Chi^2^ = 44538.17, df = 3, *p* < 0.00001, *I*^2^ = 100%. Therefore, a random-effects model was used for the analysis (Figure 5).

#### 3.3.5 Percentage change in BMD of hip joint

**Figure 6:**
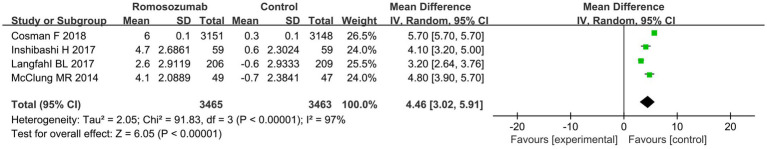
Forest plot comparing hip joint BMD between two groups after 12 months of treatment.

Percentage BMD change in the romosozumab versus control group at 12 months was evaluated by four studies. The meta-analysis results showed a significant improvement in percentage change in hip joint BMD with romosozumab compared to standard care (MD = 4.46; 95% CI = 3.02–5.91, *p* < 0.00001; *I*^2^ = 97%) (Figure 6). The heterogeneity test showed that with Chi^2^ = 91.83, df = 3, *p* < 0.00001, *I*^2^ = 97%, the results indicated that heterogeneity was evident in the research data (Figure 6).

#### 3.3.6 Percentage change in BMD of femoral neck

**Figure 7:**
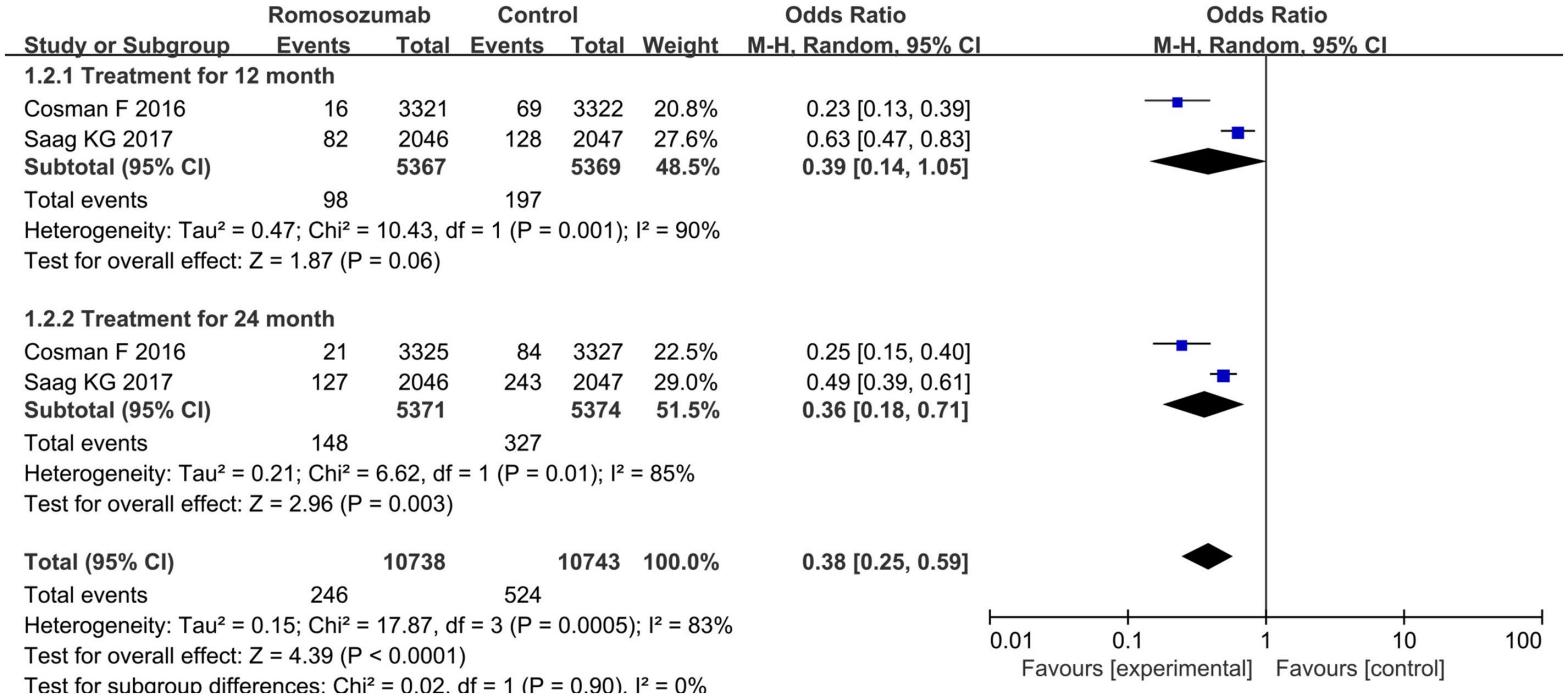
Forest plot comparing BMD of the femoral neck between two groups after 12 months of treatment.

After 12 months of treatment, four studies compared the percentage change in the BMD of the femoral neck. The pooled results showed a significant improvement in hip BMD with romosozumab (MD = 4.24; 95% CI = 2.92–5.56, *p* < 0.00001; *I*^2^ = 100%) (Figure 7). The heterogeneity test results indicated significant heterogeneity among the included studies (Chi^2^ = 3713.68, df = 3, *p* < 0.00001, *I*^2^ = 100%).

#### 3.3.7 Safety analysis

Five clinical controlled studies reported the incidence of adverse reactions (14–18). The study by Langdahl et al. (15) reported that nasopharyngitis (13% vs. 10%), hypercalcemia (1% vs. 10%), and arthralgia (10% vs. 6%) were common adverse reactions after treatment with romosozumab and standard care. In terms of adverse reactions, there was no notable difference (*p* > 0.05). Study by Ishibashi et al. (16) reported that patients treated with romosozumab had a higher risk of severe cardiovascular adverse reactions.

#### 3.3.8 Publication bias analysis

Funnel plots were drawn based on vertebral fracture rate, non-vertebral fracture rate, and clinical fracture rate. Publication bias was analyzed (Supplementary Figures S3–S5). Upon analyzing the funnel plots, the majority showed symmetrical distribution.

## 4 Discussion

PMOP is a subtle disease that often presents no symptoms in the initial stages. It is characterized by a reduction in BMD and microstructural changes in the bone, which can result in decreased bone strength and a higher likelihood of fractures (20). The main factor influencing postmenopausal bone mass loss is estrogen deficiency. The decrease in estrogen during menopause can induce an increase in RANK receptor expression on the osteoclast membrane via the RANKL pathway during osteoclast differentiation, as well as a decrease in the secretion of osteoprotegerin (OPG). Within the RANKL pathway, RANKL is expressed by osteoblasts and bone marrow stromal cells, which then bind to RANK receptors on the surface of osteoclast precursors. This binding promotes osteoclast differentiation and increases bone resorption. This imbalance directly leads to rapid bone loss and an increased risk of osteoporotic fractures. Because of the high incidence of OP and the severe consequences of osteoporotic fractures, the WHO defines OP as a major public health problem of concern (21). It is estimated that 50-year-old women have a 50% chance of experiencing osteoporotic fractures. OP can lead to a significant reduction in their quality of life. Treating osteoporotic fractures can also place a huge burden on the entire healthcare system, and this burden will increase as the elderly population grows each year (22).

The current study demonstrated a significant reduction in the incidence of vertebral fractures at 12 and 24 months, as well as a decrease in nonvertebral and clinical fractures, with romosozumab compared to standard therapy. Supporting our findings, a meta-analysis by Liu et al. (23) also found that romosozumab was associated with a significantly reduced risk of new vertebral fracture, nonvertebral fractures and hip fractures at 24 months. Studies on the population of Latin American countries show that the incidence of osteoporosis (OP) varies from 22.2 to 33.8% due to differences in sample size, sample screening criteria, and research methods. The incidence of osteoporotic brittle fractures caused by frequent falls is as high as 11 to 23.8% (24). Among them, the mortality rate also increased remarkably with the increase of physical activity disorder and life quality degeneration, ranging from 21.5 to 30% (25). In Latin American countries, each post-menopausal patient with OP spends approximately US$775 per year on OP treatment, and the average cost of an 11-day conservative hospital stay for hip fracture in a Latin American public hospital is approximately US$394,000, with a mortality rate of 23.3% after 6 months (26). Currently, the representative drug for promoting bone resorption is tropism, but its widespread clinical use is limited by the increased risk of osteosarcoma (27). As a monoclonal antibody, romosozumab exerts its anti-osteoporotic effect by antagonizing the activation of the Wnt signaling pathway by osteosclerotic proteins and binding to RANKL (28). According to incomplete statistics, within 1 year after the occurrence of hip fracture, about 1 to 5 patients will die of various complications, and the overall disability rate of hip fracture is as high as 50% (29). Vertebral fracture is the most common fracture type in PMOP, and the probability of recurrent fracture after a vertebral fracture is relatively high. Combined with our results, the incidences of vertebral, non-vertebral, and clinical fractures after treatment were compared and analyzed. Study participants had a remarkably lower incidence of vertebral fractures, non-vertebral fractures, and clinical fractures. Our findings on BMD improvement at the lumbar spine, total hip, and femoral neck with romosozumab at 12 months corroborate the positive effects reported by Liu’s et al. (23) meta-analysis. Combining romosozumab therapy with conventional treatment can have a synergistic effect, significantly reducing the incidence of fractures and enhancing the clinical prognosis of patients with PMOP. Diagnostics of OP are based on BMD, which is the gold standard worldwide. This study compared and analyzed the changes in BMD of the lumbar spine, hip, and femoral neck bones after 12 months of treatment. Based on our analysis, the study group had higher BMD in the lumbar spine, hip, and femoral neck bones. It is revealed that the long-term effect of romosozumab combined with routine treatment is better and can successfully enhance the BMD of PMOP patients, promote bone strength, and significantly reduce the risk of fractures. The reason is that romosozumab, as a monoclonal antibody, can activate the Wnt pathway by antagonizing sclerostin, ensuring the normal transmission of the Wnt/β-catenin pathway, promoting bone formation, and inhibiting bone resorption (30, 31).

Cardiovascular events have been the focus of various drug studies, and romosozumab is no exception. The cardiovascular effects of sclerostin, the target of romosozumab, are complex, and changes in this index alone cannot be used to explain cardiovascular events. Additionally, the Wnt signaling pathway has both advantages and disadvantages regarding cardiovascular disease, with more evidence suggesting that this pathway has a protective effect. A black box warning has been issued for romosozumab in patients with high risk factors for cardiovascular disease or stroke. A study by Saag et al. (17) indicated that the rate of serious cardiovascular events was higher with romosozumab than with alendronate. The incidence of adverse reactions to romosozumab was similar to that of the control group. The mean age of the patients contained by McClung et al. (18) was higher; on the one hand, older patients have a poorer cardiovascular base; on the other hand, the effect of romosozumab on osteosclerotic proteins and the Wnt pathway may be influenced by age. Alendronate has a protective effect on the cardiovascular system, but this has not been confirmed by the meta-analysis. This study included a small number of studies and did not classify cardiovascular events. The number of one-off studies was low due to the lack of original reports. Additionally, there were ethnic and gender differences in the study’s population, which did not facilitate the generalization of the findings. Most of the study cycles focused on 12 months, and the evaluation of efficacy and safety was limited due to the small number of original studies and the lack of stratified analysis for different treatment cycles and long-term outcomes. This study has several limitations. Firstly, a small number of studies were included, and most of these were conducted in Western countries, which limits the generalizability of the findings to other races and regions. Secondly, the meta-analysis was limited to studies in English, which may lead to publication bias. Finally, due to the limited number of studies, we did not perform a sensitivity analysis, which may influence the quality of the results. Therefore, validation will require more high-quality RCTs.

## 5 Conclusion

In conclusion, the combination of romosozumab and conventional therapy emerges as a viable clinical treatment option for postmenopausal patients with OP. Our results demonstrated a significant reduction in fracture risk and improvement in BMD among postmenopausal women with OP who received this treatment, with no notable increase in the incidence of adverse effects.

## Data Availability

The original contributions presented in the study are included in the article/Supplementary material, further inquiries can be directed to the corresponding author.
