# Supplementary material for: Effects of romosozumab combined with routine therapy on pain relief, disease progression and adverse reactions in patients with postmenopausal osteoporosis: a systematic review and meta-analysis
Source: Front Med (Lausanne). 2024 Aug 14;11:1440948. doi: 10.3389/fmed.2024.1440948 (PMC11349545; doi:10.3389/fmed.2024.1440948)
Supplement: Supplementary file 1 [file Data_Sheet_1.docx]

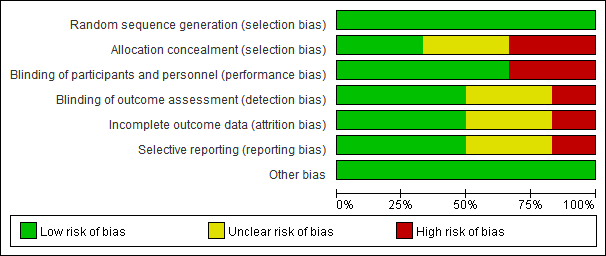


**Supplementary Figure 1. Risk bias chart**


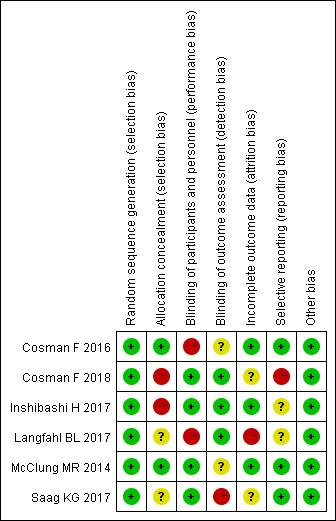


**Supplementary Figure** 2: summary chart of risk bias


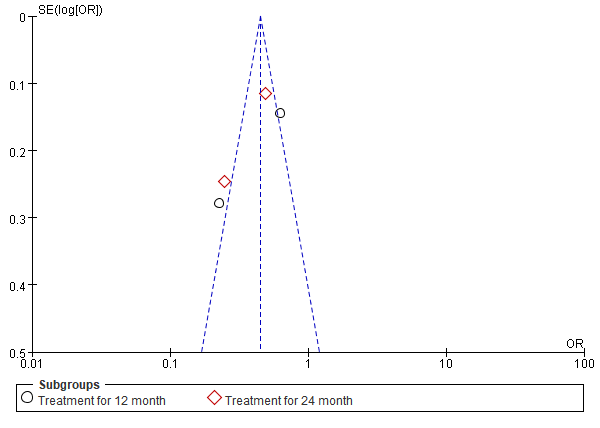


**Supplementary Figure 3. Funnel chart based on vertebral bone fracture rate**


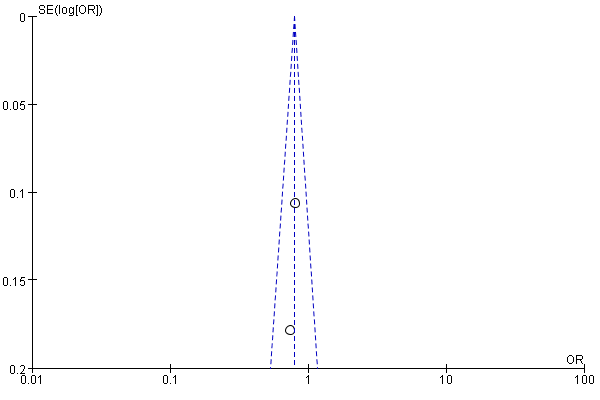


**Supplementary Figure 4.** Funnel chart based on non-vertebral bone fracture rate


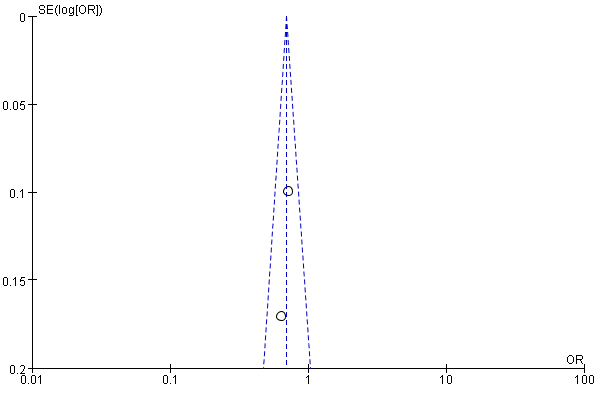


**Supplementary Figure 5.** funnel chart based on clinical bone fracture rate
